# Radioreparative Effect of Diode Laser on Leukopoiesis Recovery: A Pilot Study

**DOI:** 10.3390/life14010123

**Published:** 2024-01-15

**Authors:** Jaroslav Průcha, Zuzana Šinkorová, Anna Carrillo, Tomáš Burda, Julie Čuprová

**Affiliations:** 1 Department of Information and Communication Technologies in Medicine, Faculty of Biomedical Engineering, Czech Technical University in Prague, Sitna Sq. 3105, 272 01 Kladno, Czech Republic; prucha.jar@gmail.com (J.P.); burdato5@fbmi.cvut.cz (T.B.); 2 Department of Radiobiology, Faculty of Military Health Sciences, University of Defence, Třebešská 1575, 500 02 Hradec Králové, Czech Republic; zuzana.sinkorova@unob.cz (Z.Š.); anna.carrillo@unob.cz (A.C.); 3 Department of Healthcare and Population Protection, Faculty of Biomedical Engineering, Czech Technical University in Prague, Sitna Sq. 3105, 272 01 Kladno, Czech Republic

**Keywords:** laser therapy, leukopoiesis reparation, ionizing radiation, radiation sickness, peripheral blood examination

## Abstract

The aim of the present study was to investigate the effect of laser therapy on leukopoiesis recovery after irradiation with ionizing radiation. A dose of ionizing radiation was used that induced the hematological form of radiation sickness, reducing the number of blood cells. Subsequently, mice were treated with non-ionizing laser radiation. Based on the examination of the peripheral blood, the study found that laser therapy significantly impacted the number of eosinophils and basophils two weeks after irradiation. Laser therapy also led to the faster reparation of the lymphocyte lineage of white blood cells (WBCs). The research showed that the examined therapeutic laser had a long-term radioreparative effect on gamma-irradiated mice, improving the absolute counts of different lines of WBCs. The results of this study could have implications for the treatment of radiation sickness in humans.

## 1. Introduction

Ionizing radiation (IR) can cause on-target and off-target (bystander) effects. The communication of stress-triggering molecules from cells exposed to IR propagates the effects of stress, including oxidative stress, to surrounding cells and their progeny. The induced effects may be substantially similar to those observed in the progeny of irradiated cells [1].

Lasers are a source of non-IR and are used in various fields of science and technology. One type of laser commonly used in biomedicine is the therapeutic laser. Some of the effects of therapeutic lasers that are currently described include anti-inflammatory effects [2,3,4], analgesic effects [5,6], wound healing [7], and stimulating effects [8,9,10], as observed in in vitro and in vivo studies. The effect of therapeutic lasers in terms of reducing oxidative stress by affecting the concentrations of oxidative and antioxidant enzymes such as catalase [11,12,13] and superoxide dismutase [3,11,14] is well described in the literature. Moreover, the irradiation of tissue with a therapeutic laser leads to an increase in glutathione concentrations [15] and a decrease in the rate of lipid peroxidation [3,12,16,17]. In this way, the laser can be used to influence oxidative processes in the tissue of irradiated organisms and thus reduce the adverse effects of IR. An important direction of work, both for other authors [18,19] and for our collective, is the investigation of the effect of lasers on leukopoietic recovery, which helps to monitor the immune status of the organism, including the possibility of increasing the survival rates of experimental animals after gamma irradiation.

In our previous experiment, a 940 nm, 5 W, 20 Hz, 3 J/cm^2^ energy density laser was shown to prolong the lifespan of mice exposed to a lethal dose of gamma radiation (8.7 Gy) when used 24 h after gamma irradiation. In addition, laser therapy with the above parameters increased the absolute numbers of leukocytes (LEUs), including granulocytes, lymphocytes (LYMs), and T-lymphocytes (T-LYMs), in the peripheral blood by 1.5–3.67 times on day 12, compared to the group of gamma-irradiated animals [20].

In the present experiment, we decided to investigate the dynamics of changes after the irradiation of mice with IR and a laser. To investigate leukopoietic recovery, blood was collected in the 2nd, 4th, and 8th weeks after irradiation with IR.

## 2. Materials and Methods

### 2.1. Experimental Animals

A total of 65 2-month-old female mice, inbred strain C57BL/6 (Velaz, s.r.o., Únětice, Czech Republic) with an initial body weight of 20 ± 1 g, were used in the study. The animals were housed in standard vivarium caging under controlled conditions (temperature, pressure) and a 12/12 light/dark cycle. Food and water were provided ad libitum. The mice were acclimated for 1 week before use. All experiments were approved by the Ethics Committee of the Faculty of Military Health Sciences in Hradec Kralove, Czechia and by the Ethics Committee of the Ministry of Defense of the Czech Republic.

### 2.2. Groups

Following an acclimation period, the mice were randomized into 3 experimental groups. The mice in group A (control, *n* = 11) were in their cages for the entire duration. Group A laboratory animals were not exposed to IR or laser therapy but were kept under the same conditions as other groups of mice. In group B, the mice (*n* = 27) were exposed to gamma irradiation only (see Section 2.4). In group C (*n* = 27), the mice were exposed to gamma irradiation and laser radiation (see Section 2.4 and Section 2.5). We chose 3 time points for blood analysis: 14, 28, and 56 days after IR. Nine mice each from groups B and C were euthanized by narcotic gas overdosing at each time point. Their peripheral blood was collected intracardially into a heparin-containing solution (final concentration 20 international units (UI)/mL, Zentiva, Prague, Czech Republic). The blood of mice from group A was analyzed on day 14. A summary of the distribution of animals in the study groups can be seen in Table 1.

### 2.3. Source of Gamma Rays

A 60Co gamma-ray source (Chisocobalt, Chirana Technik, Roznov pod Radhostem, Czech Republic) was used. The geometry of the exposure system was verified using an ionizing chamber dosimeter, type VAJ 27012 (RFT Klinishes Dosimeter 27012, Veb Roboton Messelektronik ‘Otto Schon’, Dresden, Germany).

### 2.4. Gamma Irradiation

Unanesthetized mice from experimental groups B and C were individually immobilized in special cages, which were placed in a circular layout (called a “carousel”). Before irradiation, all mice from experimental groups B and C were confined to the irradiation drum in separate compartments. Subsequently, the drum containing the laboratory animals was placed under the irradiator so that whole-body uniform exposure to ionizing gamma radiation occurred. The mice were irradiated with a single non-lethal dose of 4 Gy (1.3 Gy/min) using a Co-60 gamma radiation source. After irradiation, the mice were immediately placed in breeding cages. The irradiated animals were randomly divided into two groups of 27. One group was left without exposure to additional physical energy (group B) and the other group (group C) was subjected to laser therapy on the day after irradiation with IR (see Section 2.5).

### 2.5. Laser Irradiation

The mice from group C, irradiated with gamma radiation, were additionally treated with non-ionizing laser radiation daily. The first procedure took place 24 h after irradiation; the other procedures were performed after 48 h. The total number of laser therapies was 5.

A special laser was constructed to perform this experiment. Its design allowed the simultaneous use of a 915 nm wavelength with power of 15 W and a 1064 nm wavelength with power of 25 W. Both wavelengths were guided by a flexible optical cable consisting of a tight bundle of multimodal light guides with a core diameter of 40 μm, type GF60, numerical aperture NA = 0.64 (corresponding to an alpha angle = 39.8°), attenuation 1.1 dB/m (manufacturer Lifatec, Hüttenberg-Rechtenbach, Germany). The diameter of the constant flux beam head was 3 mm. The head was perfectly polished, and, to avoid reflexes, it was placed directly on the skin of the animal, over the spleen area. This location was chosen because the spleen is an important organ in the mouse body in terms of hematopoiesis and is more accessible than other hematopoietic organs. The laser radiation was applied in pulsed mode. The pulse duration was 250 microseconds, and the pulse frequency was 48 Hz. A total of 5 treatments were administered during the first two weeks after the application of IR. The last treatment occurred 2 days before the first test. The total laser energy generated was 300 J. During irradiation, the mice were in the dorsal position. Anesthesia was not performed during laser therapy.

The calculated values of the physical quantities mediating the interaction of laser radiation with living matter are described in Table 2.

### 2.6. Peripheral Blood

We collected the peripheral blood of the mice intracardially into a heparin-containing solution (final concentration 20 UI/mL, Zentiva, Prague, Czech Republic), with the animal under anesthesia, on days 14, 28, and 56 after gamma radiation. Total white blood cells (WBCs), absolute values of LYMs, monocytes (MONs), neutrophils (NEUs), eosinophils (EOSs), and basophils (BASs) were determined using the Sysmex XE-2100 hematological analyzer (Sysmex-Toa, Kobe, Japan).

Flow cytometry was used to determine the relative numbers of T- and B-lymphocytes (B-LYMs) and natural killer (NK) cells in peripheral blood. The surface immunophenotyping of cells was performed using monoclonal antibodies (BD Biosciences, Allschwil, Switzerland):

1. PE Rat Anti-Mouse CD3 Molecular Complex—for the labeling of T-lymphocytes;
2. PerCP Rat Anti-Mouse CD45R/B220—for the labeling of B-lymphocytes;
3. PE-Cy™7 Mouse Anti-Mouse NK-1.1—for the labeling of NK cells.

A cocktail of antibodies was added to 50 μL of whole blood from the laboratory animals (see Table 3). This was followed by 20 min incubation of the mixture (dark, 4 °C). After this time, 1 mL of Easy-Lyse Lysing Reagent (Agilent DAKO, Prague, Czech Republic) was added to the mixture to induce the lysis of erythrocytes. The solution was prepared by 1:9 dilution with deionized water. The incubation of the peripheral blood with the lysis solution lasted five minutes at room temperature. Subsequently, the samples were washed 2 times with phosphate-buffered solution (phosphate solution with 0.15 M NaCl), followed by centrifugation (5 min, 1600 rpm, 8 °C). After centrifugation, the supernatant was washed, and the sediment was vortexed. The wash solution was added to the sediment to give a final volume of approximately 100 pl. The labeled samples were analyzed by flow cytometry. Shortly before measurement, propidium iodide solution at a concentration of 0.1 pg/mL was added to the samples to exclude dead cells from the analysis.

Multicolor immunophenotyping analysis of the main LYM populations was performed with a flow cytometer (CyAn, Beckman Coulter, Prague, Czech Republic), excluding doublets and multiplets by using the gating strategy of forward scatter (FSC)/side scatter (SSC) in dotplots and excluding dead cells (propidium-iodide-positive) from the analysis. At least 10^5^ live cells were included in each analysis.

Next, LYMs (100%) were divided into subpopulations based on the expression of surface features characteristic of the main LYM population. Then, the relative percentages of T- and B-LYMs and NK cells were then determined by analysis and their absolute numbers (from the values of total numbers of lymphocytes per 1 mL from the hematological analyzer) were recalculated for each experimental animal.

### 2.7. Data Analysis

The IBM SPSS Statistics v21.0 program was chosen for statistical data processing. The normality of the data was verified using the Kolmogorov-Smirnov test. Data were expressed as mean and standard deviation. One-way ANOVA with Tukey post hoc tests was applied to evaluate differences between groups. A *p* < 0.05 was considered indicative of statistical significance.

## 3. Results

### 3.1. Absolute WBC Count in Control Mice (Group A), Mice Treated with Gamma Radiation (Group B), and Irradiation Group with Additional Laser Treatment (Group C)

**Figure 1:**
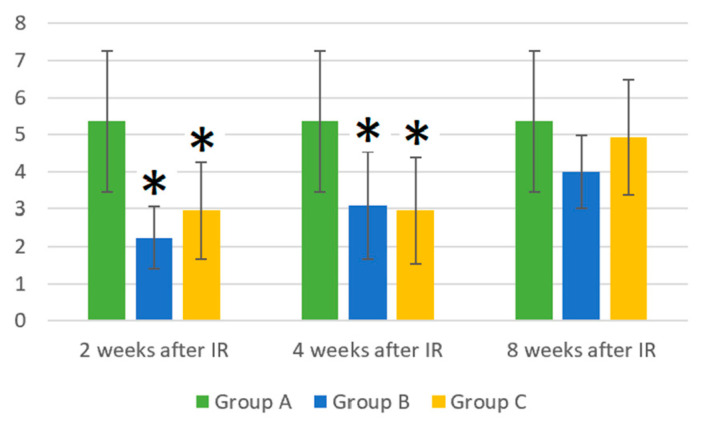
Absolute counts of white blood cells (×10^6^/mL). Data are shown as mean ± SD. * indicates *p* < 0.05, when experimental group B or C was compared with control group A. Group A is a control group of healthy mice, group B contains mice irradiated with gamma irradiation only, group C contains mice irradiated with a laser after gamma irradiation.

At 2 and 4 weeks after the gamma irradiation of the mice, there was a statistically significant decrease in the number of WBCs in the peripheral blood in groups B and C compared to the control group A (*p* < 0.05). The measured values are shown in Figure 1.

### 3.2. Dynamic Changes in the LYM Count of Control Mice (Group A), Mice Treated with Gamma Radiation (Group B), and the Irradiation Group with Additional Laser Treatment (Group C)

**Figure 2:**
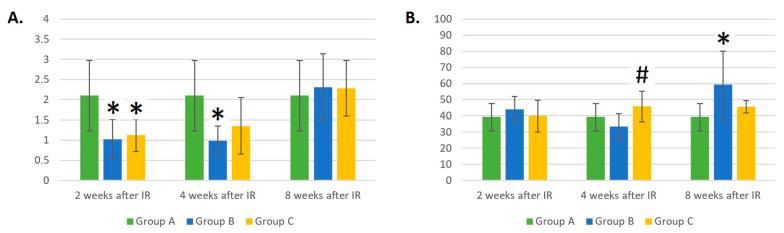
(**A**) Absolute counts of lymphocytes (×10^6^/mL). (**B**) Relative counts of lymphocytes (%). Data are shown as mean ± SD. * indicates *p* < 0.05, when experimental group B or C was compared with control group A. # indicates *p* < 0.05, when experimental group B was compared with experimental group C. Group A is a control group of healthy mice, group B contains mice irradiated with gamma irradiation only, group C contains mice irradiated with a laser after gamma irradiation.

The absolute number of LYMs was two times lower 2 weeks after IR in both experimental groups compared to the control group A (*p* < 0.05 for both groups), which was expected. However, at 4 and 8 weeks after gamma irradiation, a trend towards the faster recovery of LYM counts could be seen in the group of mice irradiated with a laser (group C, *p* = 0.09) than in the group not irradiated with a laser (group B, *p* < 0.05). Nevertheless, 8 weeks later, IR groups B and C did not differ from control group A in the absolute count of LYMs (Figure 2A).

Four weeks after IR, the relative count of LYMs in the group of mice irradiated with a laser (group C) was 37% higher than in the group where the laser was not applied (group B) (*p* < 0.05). Eight weeks after IR, the relative count of LYMs in group B was 51% higher compared to that in group A (*p* < 0.05). The dynamic changes in the relative counts of LYMs may be seen in Figure 2B.

### 3.3. Dynamic Changes in B- and T-Cell and NK Counts in Control Mice (Group A), Mice Treated with Gamma Radiation (Group B), and the Irradiation Group with Additional Laser Treatment (Group C)

**Figure 3:**
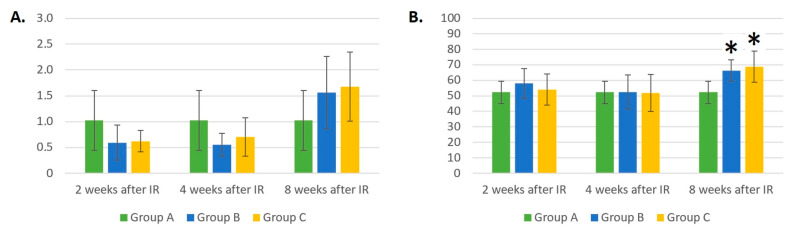
(**A**) Absolute counts of B-lymphocytes (×10^6^/mL). (**B**) Relative counts of B-lymphocytes (%). Data are shown as mean ± SD. * indicates *p* < 0.05, when experimental group B or C was compared with control group A. Group A is a control group of healthy mice, group B contains mice irradiated with gamma irradiation only, group C contains mice irradiated with a laser after gamma irradiation.

At week 8 after IR, we observed a trend toward an increased absolute count of B-lymphocytes in groups B and C compared to the healthy mice from group A (Figure 3A).

The relative number of B-LYMs in groups B and C was statistically (*p* < 0.05) significantly different compared to group A only 8 weeks after gamma irradiation (Figure 3B). At this time point, groups B and C had 27% and 32% higher relative counts of B-LYMs than group A.

**Figure 4:**
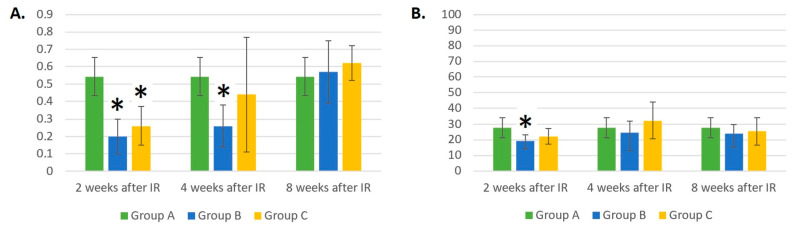
(**A**) Absolute counts of T-lymphocytes (×10^6^/mL). (**B**) Relative counts of T-lymphocytes (%). Data are shown as mean ± SD. * indicates *p* < 0.05, when experimental group B or C was compared with control group A. Group A is a control group of healthy mice, group B contains mice irradiated with gamma irradiation only, group C contains mice irradiated with a laser after gamma irradiation.

The absolute count of T-LYMs (Figure 4A) was statistically significantly different between the non-IR and IR-treated groups 2 weeks after gamma irradiation. In both irradiated groups, the absolute T-LYM counts were 2–2.5 times lower than in the group of healthy mice (*p* < 0.05). However, 4 weeks after IR, a statistically significant decrease in the absolute count of T-LYMs could be observed only in group B, composed of mice irradiated with gamma radiation (*p* < 0.05). The laser-irradiated mice in group C showed a faster increase in absolute T-LYM counts 4 weeks after gamma irradiation. Eight weeks after IR, the absolute T-LYM counts in all three groups were comparable.

The relative T-LYM count was lower (*p* < 0.05) in group B compared with group A only 2 weeks after IR irradiation (Figure 4B).

**Figure 5:**
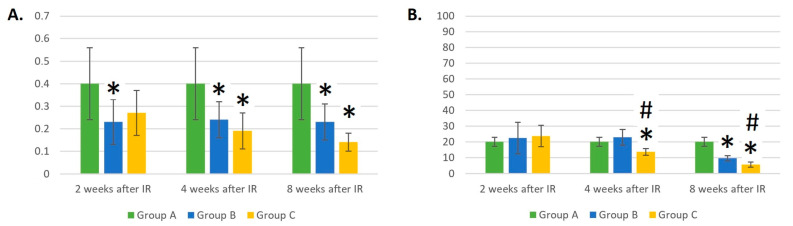
(**A**) Absolute counts of NK cells (×10^6^/mL). (**B**) Relative counts of NK cells (%). Data are shown as mean ± SD. * indicates *p* < 0.05, when experimental group B or C was compared with control group A. # indicates *p* < 0.05, when experimental group B was compared with experimental group C. Group A is a control group of healthy mice, group B contains mice irradiated with gamma irradiation only, group C contains mice irradiated with a laser after gamma irradiation.

The irradiation of mice with IR and laser light led to statistically significant reductions in NK cell numbers at different time points compared to control group A. From Figure 5A, it can be observed that additional laser therapy decreased the absolute counts of NK cells to a greater extent than gamma irradiation. By week 8 after irradiation, the number of NK cells in group C was reduced by two and three times compared to groups B and A, respectively (*p* < 0.05).

At week 4 after IR, a decrease in the relative number of NK cells could be seen in the laser-irradiated group compared to both the healthy mice in group A and the group of mice irradiated with ionizing radiation only (*p* < 0.05). At week 8 after IR, there was a decrease in the relative number of NK cells in groups B and C compared to group A (*p* < 0.05). At this time point, in group C, the relative number of NK cells was 67% lower compared to group B containing gamma-irradiated mice (*p* < 0.05). A trend was seen wherein the laser therapy of gamma-irradiated mice likely led to an even more drastic reduction in the absolute and relative counts of NK cells than in group B, containing mice irradiated with IR only.

### 3.4. Dynamic Changes in MON Count in Control Mice (Group A), Mice Treated with Gamma Radiation (Group B), and the Irradiation Group with Additional Laser Treatment (Group C)

**Figure 6:**
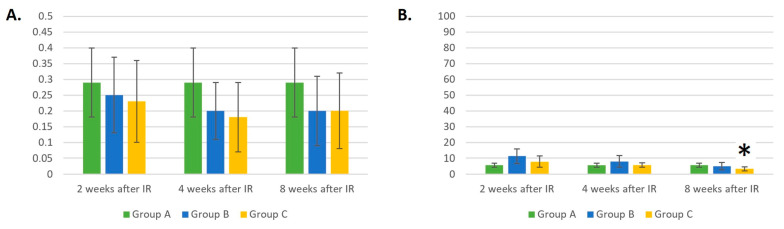
(**A**) Absolute counts of monocytes (×10^6^/mL). (**B**) Relative counts of monocytes (%). Data are shown as mean ± SD. * indicates *p* < 0.05, when experimental group B or C was compared with control group A. Group A is a control group of healthy mice, group B contains mice irradiated with gamma irradiation only, group C contains mice irradiated with a laser after gamma irradiation.

The absolute count of MONs was not significantly affected by IR exposure and remained approximately the same throughout the experiment (Figure 6A). Laser therapy influenced the absolute count of MONs 4 weeks after IR. At this time point, there were fewer MONs in group C than in control group A (*p* = 0.083).

Eight weeks after IR, the relative number of MONs in the laser-irradiated group C was almost two times lower compared to the control group A (*p* < 0.05). This result may be seen in Figure 6B.

### 3.5. Dynamic Changes in the NEU Count in Control Mice (Group A), Mice Treated with Gamma Radiation (Group B), and the Irradiation Group with Additional Laser Treatment (Group C)

**Figure 7:**
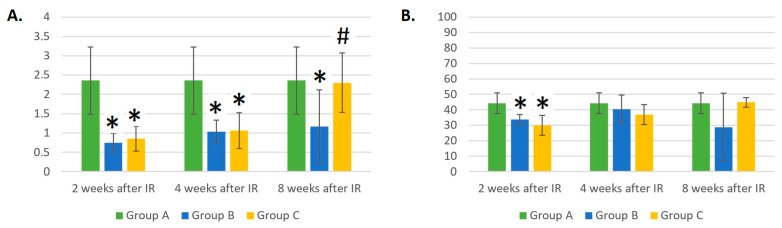
(**A**) Absolute counts of neutrophils (×10^6^/mL). (**B**) Relative counts of neutrophils (%). Data are shown as mean ± SD. * indicates *p* < 0.05, when experimental group B or C was compared with control group A. # indicates *p* < 0.05, when experimental group B was compared with experimental group C. Group A is a control group of healthy mice, group B contains mice irradiated with gamma irradiation only, group C contains mice irradiated with a laser after gamma irradiation.

As expected, the absolute number of NEUs (Figure 7A) was statistically significantly lower 2 weeks after irradiation in groups B and C compared to the control group A (*p* < 0.01). As early as 4 weeks after irradiation, an increase in NEU counts was evident in both groups B and C compared to the results from the previous 2 weeks. However, the number of NEUs remained lower in groups B and C compared to the control group (*p* < 0.05). Eight weeks after the gamma irradiation of mice, an increase in the number of NEUs in group C was evident. The results did not differ compared to the control group, but they were statistically significantly different compared to group B (*p* < 0.05).

Two weeks after gamma irradiation, the relative number of NEUs (Figure 7B) was lower in the blood of both groups B and C compared to the control group (*p* < 0.05), which was to be expected. However, there was a trend toward a steady increase in the relative count of NEUs in group C, which, after 4 weeks, no longer differed from control group A. In group B, changes in the percentage of NEUs were highly variable. For example, 4 weeks after gamma irradiation, the percentage of NEUs did not differ from that in control group A, but 8 weeks after irradiation, a trend toward a decrease in the percentage of NEUs (*p* = 0.068) and large variability in the measured values (dispersion of results) could be seen.

### 3.6. Dynamic Changes in the Eosinophil and Basophil Counts in Control Mice (Group A), Mice Treated with Gamma Radiation (Group B), and the Irradiation Group with Additional Laser Treatment (Group C)

**Figure 8:**
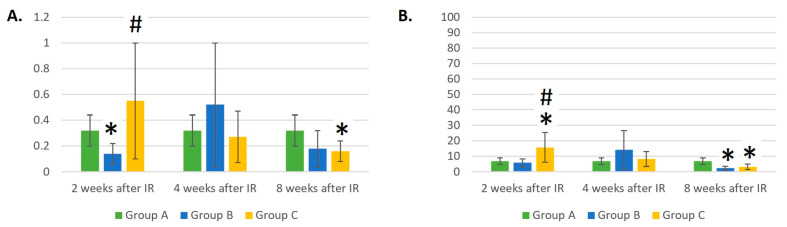
(**A**) Absolute counts of eosinophils (×10^6^/mL). (**B**) Relative counts of eosinophils (%). Data are shown as mean ± SD. * indicates *p* < 0.05, when experimental group B or C was compared with control group A. # indicates *p* < 0.05, when experimental group B was compared with experimental group C. Group A is a control group of healthy mice, group B contains mice irradiated with gamma irradiation only, group C contains mice irradiated with a laser after gamma irradiation.

**Figure 9:**
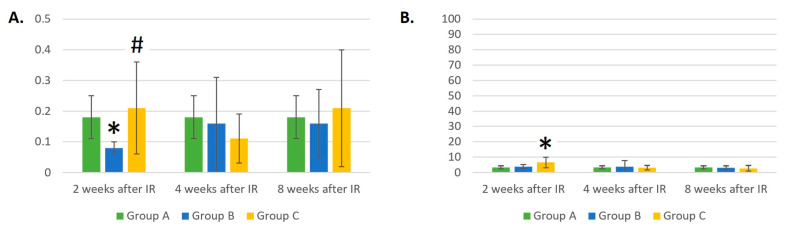
(**A**) Absolute counts of basophils (×10^6^/mL). (**B**) Relative counts of basophils (%). Data are shown as mean ± SD. * indicates *p* < 0.05, when experimental group B or C was compared with control group A. # indicates *p* < 0.05, when experimental group B was compared with experimental group C. Group A is a control group of healthy mice, group B contains mice irradiated with gamma irradiation only, group C contains mice irradiated with a laser after gamma irradiation.

Two weeks after IR, the EOS and BAS counts were statistically lower in group B compared to control group A (*p* < 0.05). Moreover, 4 and 8 weeks after IR, the numbers of EOSs and BASs showed strong dispersion, which may be seen in Figure 8A and Figure 9A. The absolute numbers of EOSs (Figure 8A) and BASs (Figure 9A) were almost 4 and 2.6 times higher 2 weeks after IR in group C compared to group B (*p* < 0.05). At this time point, the number of EOSs was 1.7 times higher in group C compared to group A, but the result was not statistically different due to the large variability in the measured values. However, at 4 and 8 weeks after IR, we saw a decrease in the absolute number of EOSs in group C compared to group A; at the last time point, there was an almost two-times-lower number of EOSs in group C compared to group A (*p* < 0.05).

The percentage representation of EOSs (Figure 8A) and BASs (Figure 9B) confirms the results of our experiment [21]. Two weeks after IR, laser therapy in group C led to an increase in the percentages of EOSs and BASs compared to control group A and group B (*p* < 0.05 and *p* = 0.053). Eight weeks after IR, there was an almost two-times-lower relative number of EOSs and BASs in groups B (*p* > 0.05) and C compared to group A (*p* < 0.05). Strong variability in the results was also seen in the relative numbers of EOSs and BASs throughout the experimental period.

In our previous experiment [21], we observed mice irradiated with a lethal dose of gamma radiation and laser therapy performed only once, but the trend of an initial increase in the EOS and BAS parameters could be observed with lower doses and several laser therapy treatments in the more recent experiment. We could not determine how the absolute and relative counts of EOSs and BASs changed after 2 weeks from the beginning of the experiment. Here, we observed that the initial increase was reduced one month after irradiation. The decrease was more intense in the case of EOSs.

## 4. Discussion

In the present study, we investigated the effect of laser therapy (915 (15 W) and 1064 nm (25 W), pulsed mode with frequency of 48 Hz, with a total energy density of 4285 J/cm^2^ after five procedures) on leukopoiesis recovery after irradiation with a single non-lethal dose of gamma radiation (4 Gy). From the published results, it is apparent that the examined therapeutic laser had a long-term radioreparative effect in mice irradiated by gamma irradiation. A non-lethal dose of IR significantly and continually affected the relative and absolute counts of different lines of WBCs compared to the data obtained from the control group. As long as gamma-irradiated mice were treated with laser therapy, there was a trend toward an increased number of WBCs and absolute and relative counts of NEUs (*p* = 0.043 and 0.055, respectively) in their peripheral blood 8 weeks after the beginning of the experiment, compared to the gamma-irradiated mice. Eight weeks after IR, the absolute number of NEUs was not statistically different compared to healthy mice from group A against group B (*p* = 0.036). On the other hand, laser therapy affected the lines of NK cells, and their absolute and relative counts were lower than in group B, i.e., in mice irradiated with gamma radiation only. The greatest impact was seen 2 weeks after IR. The greatest effect of laser therapy on the absolute numbers of LEU subpopulations was apparent in EOS and BAS. As a result of this impact, the mice in group C had a greater number of WBCs than the mice in group B. This effect of laser therapy corresponded with results from our previous experiment [20] and may have led to the more effective regeneration of hematopoiesis and the prolonged lifespan of mice even after irradiation with a lethal dose of gamma radiation.

In our experiment, we used a dose of IR of 4 Gy, which induces the hematological form of radiation sickness in the irradiated organism. This form of radiation sickness is characterized by a reduction in the number of peripheral blood cells. This was observed in our experiment. In our previous work [20], we investigated the effect of the order of irradiation by a 940 nm laser and the influence of its energy density on the survival and recovery of hematopoiesis in mice irradiated with a lethal dose of IR. It was shown that a single laser treatment of the gamma-irradiated mice (3 J/cm^2^, 20 Hz, and 5 W) resulted in survival and lifespan extensions and statistically significantly, 2.33 to 3.33 times, higher WBC, LYM, and NEU counts on day 12 after gamma irradiation, compared to mice that did not receive the laser therapy. It was found that there was a large increase in the counts of EOSs and BASs [21] in the peripheral blood of gamma-irradiated mice that received laser irradiation. Following this study, an experiment was carried out in which we decided to look at the dynamics of the changes in peripheral blood cell numbers. In contrast to the previous experiment, we chose a different type of high-power therapeutic laser with wavelengths of 915 (15 W) and 1064 nm (10 W), a frequency of 48 Hz, and a total energy of 300 J. We performed laser therapy five times in total. We irradiated the mice with a non-lethal whole-body energy of 300 J (which represents a dose of 4285 J/cm^2^) to prevent early death and to observe the target dynamics of blood cell changes in the peripheral blood. In contrast to the previous experiment, we focused the laser beam only on the spleen region, and the laser light was applied directly on the skin of the animals. Comparing these two experiments, we can observe a common trend in the effect of Yelaser therapy on hematopoietic recovery in gamma-irradiated mice. In both experiments, WBC counts were higher in the laser-irradiated mice than in the non-laser group B. In the current experiment, the increase was 33%. WBC counts were also 24% higher 8 weeks after IR when comparing the two groups.

The increased absolute numbers of whole blood cells after exposure to gamma rays and following the application of laser treatment is strongly dependent on the different radioresistances of individual blood populations. The first increase in WBC (2 weeks after irradiation) relates to a huge proinflammatory response of the organism (increases in NEUs from organ reserves). The positive treatment effect on the main LYM population is slow and is strongly dependent on the different radiosensitivities of the main LYM population. While B-LYMs are the most sensitive to IZ, T-LYMs and NK cells are relatively radioresistant. Activation of the proliferation and differentiation of hematopoietic stem cells in the bone marrow could be induced specifically through laser treatment by activation of the differentiation of hematological stem and progenitor cells. A well-known effect of laser radiation is the stimulation of the mitotic activity of cells too [22,23]. One of the mechanisms of this laser stimulation is influence through the production of cytokines, which can stimulate cells to mitotic division and differentiation [24]. This mechanism could affect the white blood cell counts after ionizing radiation. We did not find studies in the literature evaluating the effect of laser radiation on the production of cytokines (activators of blood formation) in gamma-irradiated organisms or cells. But we hope to investigate this in future studies.

In the current experiment, laser therapy did not affect the absolute LYM count as significantly as in the previous experiment. Two weeks after irradiation, the LYM count in group C was only 10% higher than in group B. The increase was more evident in the 4th week after irradiation and was 38%. However, by week 8 post-irradiation, the absolute numbers of LYMs in both groups were already equal and were not statistically different from those in the control group. Regardless, we consider the trend of a potential increase in LYM numbers 4 weeks after irradiation to be beneficial, as one of the reasons for death in animals exposed to IR is an infection, and LYMs can prevent the spread and development of infection.

Other WBCs, namely, NEUs, have a similar role. They belong to the first line of defense against pathogens and are involved in the protection of organisms even earlier than LYMs [25]. In a previous experiment, we observed a statistically significant threefold increase in the absolute number of NEUs as early as day 12 after the irradiation of mice with IR and a laser, compared to a group of gamma-irradiated mice. In this experiment, as with LYMs, an increase could be observed up to 8 weeks after the irradiation of mice with IR. In group C, the absolute number of NEUs was essentially two times higher than in group B (*p* < 0.05). In radiobiology, granulocyte (especially NEUs) and LYM counts are used for prognostic purposes. As mentioned earlier, in the mammalian organism, granulocytes and LYMs are involved in the protection of the organism against pathogens, which, by their action on the irradiated organism, can lead to a lethal outcome. In our previous experiment [20], we observed two groups of mice irradiated with gamma radiation and a laser. The absolute numbers of NEUs and LYMs were higher than in the other groups. We hypothesized that the mice in these groups would have a better prognosis and lifespan. However, on day 30, the surviving mice were only present in one of the groups. From the comparison of the two groups, they differed significantly in only the number of EOSs and BASs [21]. In the present experiment, the same trend could be seen. The absolute numbers of EOSs and BASs were 2.9 and 1.6 times higher in group C, where the mice were irradiated with a laser, compared to group B, where the mice were irradiated with gamma radiation only. Thus, with the present experiment, we confirmed that the laser treatment of gamma-irradiated mice led to a 2–3-fold increase in EOSs, and it can be assumed that this increase could also be critical for the survival of mice after a lethal dose of IR. The previous view of EOSs as cells mainly responsible for allergic reactions and parasitic infections is outdated. EOSs are currently viewed as cells that play a key immunoregulatory role, as they are professional antigen-presenting cells, CD4+ modulators of T-LYMs, dendritic cells, B-LYMs, mast cells, NEUs, and BASs [26,27]. BASs are a population of WBCs that are involved in the protection of the body against parasites and play a role in the activation of some immunological responses (allergies, autoimmune, and inflammatory diseases) [28,29], but they are also involved in the process of immunomodulation due to the activation of TLR2 receptors, which have important antibacterial and antiviral effects [29]. In the future, it would be good to look at the effect of the rise of EOSs, BASs, and NEUs towards the kinetics of immune cells.

In Figure 6A, a trend towards a decrease in monocyte numbers can be seen after the laser irradiation of gamma-irradiated mice. Lasers are known for their reparative [7,8,9,10] and angiogenic [30,31] effect on tissues. We can assume that monocytes migrate to the tissues due to the angiogenesis and recovery taking place there [32] and do not have time to be replenished in the peripheral blood.

In our experiment, there was a decrease in the number of NK cells in the peripheral blood of mice irradiated with gamma radiation and a laser. NK cells have multiple variants in all organs and peripheral blood at one time. Some NK cell populations are resistant, and others are less resistant to the effects of laser therapy. Therefore, we think that the decrease in their number in the peripheral blood could be caused by their faster migration to the lymph nodes or could indicate a certain induced apoptotic development after the laser intervention.

From the comparison of the mean values, it can be concluded that the radioreparative effect of laser radiation is favorable; however, this was not supported by an adequate level of significance in all the expected parameters. The reason may be the small number of animals in our pilot study or the non-Gaussian distribution of data.

A slight or negative effect of laser therapy may be associated with the pro-oxidant effect of laser radiation on the body [33,34]. In our next experiments, it is necessary to measure the absorption spectra of venous blood in order to check the selected laser parameters for pro-oxidant action, and also to select the best parameters if necessary. It is also necessary to expand the experiment and investigate pro- and antioxidant molecules, and the effect of laser therapy on the hematopoietic tissue of the studied organisms.

This study has potential limitations. Our study showed that we need more mice for our experiment. There are many trend results in our paper. Due to the financial limitations of our project, we were not able to obtain more mice. We also had limitations in terms of the study animals. We could collect peripheral blood from each mouse only once. Because of this, there are three subgroups in each experimental group B and C, depending on the date of blood collection.

We did not organize a group of healthy mice irradiated with laser radiation only, because the primary aim of our study was to explore the effectiveness of laser treatment in the recovery of leukopoiesis post-irradiation and proposed that such a group would not provide direct information relevant to this specific research question. In our prior research we had already demonstrated that high-power laser treatment can reduce the counts of certain peripheral blood cells (absolute counts of white blood cells, lymphocytes, neutrophils, and monocytes) in a dose-dependent manner [20]. These fallouts helped us to explain the results in the group irradiated by gamma radiation 24 h after laser therapy [20]. In the recent study we performed laser treatment 24 h after gamma radiation and fundamental damage processes had already started, when we applied the laser therapy. Laser treatment may influence physiological processes and molecular reactions in different ways in healthy and damaged organisms. Let us look at some examples with molecules arising after exposure to ionizing radiation. Nitric oxide is an endogenous gas, which may start apoptotic processes in an organism as a result of the action of pathological factors such as ionizing radiation. Laser therapy decreased concentrations of nitric oxide in the plasma of rats with myopathy but had no effect on healthy rats [35]. Malondialdehyde is one of the final products of lipid peroxidation, which takes place in tissues due to the action of free radicals. Laser treatment inducted production of malone dialdehyde in the muscles of healthy mice, but decreased the concentration of malondialdehyde in the muscles of mice with muscle lesions [36]. Furthermore, the addition of another experimental group would require substantial additional resources (e.g., funding and time) and ethical considerations regarding the use of extra animals that would not contribute to the hypothesis (see Section 2.2).

Our study confirms that the application of the laser can be a way of supplying the energy needed for the recovery of the organism after exposure to ionizing radiation. Therefore, the primary area for using the result of our study is the faster recovery of oncological patients’ organisms after radiotherapy. Furthermore, laser therapy may be potentially utilized in persons who have been accidentally exposed to ionizing radiation (for example, after accidents at nuclear power plants).

## 5. Conclusions

In the present experiment, we investigated the dynamics of peripheral white blood cell count changes after the irradiation of mice with gamma radiation and a laser. The examined therapeutic laser had a long-term radioreparative effect on mice irradiated by gamma radiation by increasing the NEU, EOS, and BAS counts.

## Figures and Tables

**Table 1:** Description of the control and experimental groups according to the treatment performed.

Group A	Group B	Group C
Control group of mice (neither gamma nor laser irradiation)—11 mice.	Mice were irradiated with gamma irradiation ^a^ only. Tests were performed on days 14 (2 weeks), 28 (4 weeks), and 56 (8 weeks) after gamma radiation. Each group was composed of 9 mice, for a total of 27 animals.	Mice were irradiated with a laser ^b^ after gamma irradiation ^a^. Tests were performed on days 14 (2 weeks) 28 (4 weeks), and 56 (8 weeks) after gamma radiation. Each group was composed of 9 mice, for a total of 27 animals.

^a^ For more details of the gamma radiation parameters, see Section 2.3 and Section 2.4. ^b^ For more details of the laser parameters, see Section 2.5.

**Table 2:** Laser power and calculated power values on the body surface, in the target tissue, and predicted single and cumulative doses.

	Power, Radiant Flux in Pulse Amplitude	Energy Density in One Treatment (200 s)	Energy Density in the Series of 5 Procedures
Initial (0.07 cm^2^)	25 W	856 J/cm^2^	4285 J/cm^2^
Body surface (0.07 cm^2^)	15 W	514 J/cm^2^	2570 J/cm^2^
Target organ (1 cm^2^)	7.5 W	18 J/cm^2^	90 J/cm^2^

**Table 3:** Surface labeling characteristics of mouse peripheral blood.

Antibody	Fluorochrome	Emission Maximum (nm)
anti-Mouse CD3	phycoerythrin	578
anti-Mouse CD45R/B220	peridinin chlorophyll	678
anti-Mouse NK—1.1	phycoerythrin + cyanine dye	785

## Data Availability

Dataset available on request from the authors.
